# Consequences of Unexplained Experiences in the Context of Bereavement – Qualitative Analysis

**DOI:** 10.1177/00302228211053474

**Published:** 2021-12-05

**Authors:** Milla Mäkikomsi, Anja Terkamo-Moisio, Marja Kaunonen, Anna Liisa Aho

**Affiliations:** 1 Faculty of Social Sciences, 7840 Tampere University, Tampere, Finland; 2 Facylty of Health Sciences, 205537 University of Eastern Finland, Kuopio, Finland; 3 Faculty of Social Sciences, 7840 Tampere University and Pirkanmaa Hospital District, General Administration, Tampere, Finland

**Keywords:** bereavement, grief, after-death communication, continuing bonds, extrasensory perception, interpersonal relations, social relationships

## Abstract

Unexplained experiences are common among bereaved people and are a natural part of grieving, but their consequences may affect their coping with grief. However, professionals lack awareness of these unexplained experiences, which may lead to an unnecessary pathologising of the experiences and a lack of opportunity for the bereaved to process their experiences in a safe environment. The study involved an inductive content analysis of 408 narratives of the consequences of unexplained experiences shared by 181 bereaved individuals. The consequences of the unexplained experiences were: (1) Experiencing after-effects which may alleviate or aggravate wellbeing, as well as be life-affecting; (2) consequences related to sharing or concealing the experiences, and the reactions of others to recounting the experience; (3) documenting the experience through videography, photography and keeping mementos. In conclusion, these experiences have consequences to bereaved which needs to be taken account in support interventions aimed at bereaved individuals.

Unexplained experiences related to the death of a close one are common (Keen et al., 2013; Root & Exline, 2014; Steffen & Coyle, 2010). These unexplained experiences can range from extraordinary nature-related experiences such as seeing an unusual fog, to getting symbolic messages from the deceased, or feeling the presence of the deceased through various senses (Keen et al., 2013). While the cultural normativity of having such experiences varies greatly, they occur universally among different cultures (Thomson & Jaque, 2014).

According to the continuing bonds (CB) theory, such experiences can be regarded as one form of manifestation of a continuing bond with the deceased (Steffen & Coyle, 2010). A continuing bond is defined as an ongoing internal relationship between the bereaved and the deceased (Root & Exline, 2014). CB theory explains that the meaningful relationship between people does not end, but rather changes with the death of a close one (Klass & Steffen, 2018). Through grief and different kinds of formal and unformal rituals, the deceased is both detached and re-integrated into the life of the bereaved in new ways (Mathijssen, 2018). Experiencing CBs with the deceased is widely observed as a part of ordinary grief, and as such, their unnecessary pathologising should be avoided (Jahn & Spencer-Thomas, 2014; Klass & Steffen, 2018). Continuing bonds with the deceased are as complex and intersubjective as bonds between living individuals, rather than being able to be reduced to more narrow mental structures like merely a feeling or a thought (Klass & Steffen, 2018). Because post-death relationships are similar to other personal relationships, they are not unequivocally either beneficial or harmful to the bereaved (Foster et al., 2011; Klass & Steffen, 2018).

CB can manifest in a variety of ways. Unexplained experiences related to the death of a close one represent an ongoing continuing bond, but the bond can also manifest by way of cherishing the past relationship, for example, by keeping mementos or sharing memories of the deceased with others (Foster et al., 2011; Root & Exline, 2014; Suhail et al., 2011). CB manifestations can also be experienced either internally based or externalised, that is, experienced inside or outside one’s body and mind. Unexplained experiences related to the death of a close one represent both of these categories as they can range from something such as an inner feeling of communicating with the deceased, to a shared experience of seeing the form of the deceased (Root & Exline, 2014).

Even though unexplained experiences related to the death of a close one are only one form of CB manifestation, they are common among bereaved people and feature in most studies concerning CB. While such experiences often initially evoke confusion and fear in the bereaved (Keen et al., 2013), they are usually regarded as positive experiences later on (Beischel et al., 2014; Field et al., 2013; Foster et al., 2011; Jahn & Spencer-Thomas, 2014; Steffen & Coyle, 2011). The evidence of the benefits of CB manifestations for mental wellbeing is variable (Root & Exline, 2014; Wood et al., 2012). Qualitative studies have more often shown evidence to support the beneficiality of CB manifestations (e.g. Chapple et al., 2011; Nowatzki & Grant Kalischuk, 2009), while quantitative studies have associated CB manifestations with higher levels of anxiety and more severe grief reactions (Boulware & Bui, 2016; Currier et al., 2015; Field & Friedrichs, 2004; Sirrine et al., 2018). It is further unclear whether CB manifestations generate anxiety or whether such experiences are more common among bereaved individuals with existing levels of higher anxiety (Currier et al., 2015).

Internalised manifestations are often associated with better coping (Field et al., 2013). However, it is possible that only some internalised manifestations are beneficial to the bereaved. Specifically, internalised manifestations have been found to support coping among suicide-bereaved children only when manifestations bring them closer to those characteristics of the deceased that are not related to their suicide (Wood et al., 2012). Among children who have lost their mother, externalised manifestations have been found to be stronger predictors of anxiety than internalised manifestations (Field et al., 2013) and were more often associated with unresolved grief and the earlier stages of grief (Scholtes & Browne, 2015). Externalised manifestations are more common among individuals with an insecure attachment style (Ho et al., 2013). Violent death or causing the death of a close one also increases the probability of externalised manifestations and their negative consequences to the bereaved (Field & Filanosky, 2010). For some bereaved individuals, however, externalised manifestations may also be beneficial (Wood et al., 2012). Retaining symbolic objects related to the deceased was more beneficial to bereaved mothers than frequent visits to the graveyard (Harper et al., 2011).

CB manifestations have been found to help bereaved people to cope in different situations. As examples, in the context of sudden bereavement, CB manifestations have been found to have a positive impact on the grieving (Chapple et al., 2011). Also, in cases of spouse-to-spouse organ donation, such manifestations may alleviate the survivor-guilt experienced by widows (Woo & Chan, 2010). CB manifestations may also reduce the guilt related to the suicide of a close one (Wood et al., 2012) and sometimes cause the bereaved person to experience a closer relationship with the deceased than they did during their life (Keen et al., 2013). CB may also alleviate grief by reducing the fear that the memory of the deceased person will fade (Klass, 2014). Steffen and Coyle (2010) report that CB manifestations can support the post-traumatic growth of the bereaved individual, but for unexplained experiences related to the death of a close one to be beneficial, the successful mental relocation of the deceased is important in order to ensure that the experience does not induce anxiety related to questions of the deceased’s existence (Wood et al., 2012).

External factors may also affect the manifestation and consequences of CB experiences. For example, the bereaved individual’s attachment style and the type of relationship that existed between the bereaved and the deceased can contribute to the beneficiality of CB (Currier et al., 2015; Gassin & Lengel, 2014). A close, positive relationship before death predicts the experience of CB manifestations after death (Sirrine et al., 2018; Suhail et al., 2011). According to Rubin and Shechory-Stahl (2012), a positive pre-death relationship between parents and their sons who had died in war was linked to the parents’ higher wellbeing under long-term observation. Moreover, in the case of complex pre-death relationships, CB manifestations may at best bring closure, but they may also create a pressure to reconcile with the deceased (Root & Exline, 2014). In suicide bereavement, realistic memories predict CB manifestations with a higher beneficiality to the coping of the bereaved by increasing the understanding of the events leading to the death (Wood et al., 2012). Socially accepted manifestations are more beneficial to coping among bereaved mothers, than manifestations that carry social stigma (Harper et al., 2011). Furthermore, soothing manifestations tend to decrease grief symptoms, while distressing manifestations increase them (Field et al., 2013). The beneficiality of CB is also influenced by whether the bereaved welcomes an ongoing bond with the deceased and the degree of control that the bereaved feels towards the experiences (Root & Exline, 2014). Ultimately, the consequences of the unexplained experience related to the death of a close one are closely related to the meaning that the bereaved individuals assign to their experience (Keen et al., 2013).

Anticipatory beliefs regarding an afterlife may affect the consequences of CB manifestations, and religious or spiritual conviction may help individuals to understand such experiences (Keen et al., 2013; Steffen & Coyle, 2011). Those believing in an afterlife may experience more beneficial CB manifestations, as they will feel they offer proof of a future reunion and the deceased’s relief from suffering. While religious conviction may help the bereaved to understand the meaning of death and to accept death, it may also cause a fear that the deceased is, for example, suffering in hell or having a negative reincarnation experience (Root & Exline, 2014). However, grief will be alleviated if the bereaved person’s religious conviction offers explanations for the meaning of death and also supports them in having a continuing bond with the deceased (Suhail et al., 2011).

An unexplained experience related to the death of a close one may change one’s worldview, especially if the experience is not in accordance with one’s former understanding of life after death. Such an experience may also increase a belief in an afterlife and a reunion and reduce the fear of death (Keen et al., 2013). The bereaved individual’s outlook on life may also become more positive as a result of their experience and influence the choices they make later in life. The experience may also help them to build a new identity and improve their self-confidence (Steffen & Coyle, 2011), but it may also generate anxiety and create a fear of losing their mental health (Keen et al., 2013). As many CB manifestations vary in terms of their effects, more research is required on the phenomenon (Root & Exline, 2014).

While different kinds of manifestations of CB may be beneficial to the process of mental restructuring during bereavement (Thomson & Jaque, 2014), individuals require social support in order to process them (Steffen & Coyle, 2010). In many non-Western cultures, sharing unexplained experiences related to the death of a close one is commonplace, and they are regarded as being socially accepted (Keen et al., 2013). However, in Western cultures, bereaved persons often avoid sharing their unexplained experiences with professionals due to the worry of receiving a sceptical attitude towards their experiences (Beischel et al., 2014). Sharing may also be impeded by the fear of being ridiculed or stigmatised as having mental issues (Chapple et al., 2011; Keen et al., 2013). Concealing the experiences may undermine the coping process of the bereaved (Steffen & Coyle, 2011) and also leads to an underestimation of the prevalence of the experiences among professionals (Keen et al., 2013). Thus, the creation of a safe space for bereaved persons to share their unexplained experiences requires a particular show of respect and acceptance for their experiences (Steffen & Coyle, 2011).

## Methods

The research approach of this qualitative study is phenomenologic-hermeneutical, and the research paradigm is interpretivist. Herein, an experience is understood as a subjective and relativistic mental phenomenon that is real to the person experiencing it and which can be described and interpreted with words. The study looks to present the experiences as authentically as possible, but with an understanding that both the description of the experiences and the analyses of the data will necessarily involve interpretation (McChesney & Aldridge, 2019).

The purpose of this study is to generate knowledge on the consequences of unexplained experiences related to the death of a close one. In order to maintain a broad investigation, the study does not place any limitations on the cause of death, a type of experience, the time that has passed since the death or the role of the deceased in the bereaved person’s life. The goal of this study is to improve the interactions between bereaved individuals and professionals and to promote the development of support interventions for the bereaved by increasing the knowledge of the phenemenon.

In this study, a bereaved person is defined as a person whose close one has died. Additionally, this study defines coping as learning to live with grief and loss, but does not necessarily mean accepting them. As a final definition, an unexplained experience is defined as any experience the bereaved has felt to be unexplained and related to the death of a close one. The term ‘unexplained experiences’ was deliberately chosen for use in this study in order to decrease the stigmatising effect of these experiences, even though it is not widely used in literature. Specifically, the authors felt that using more commonly used terms such as ‘supernatural’ or ‘paranormal’ might lead the reader to think that the phenomenon is not ‘natural’ or ‘normal’ and by that increase the stigmatising effect of these experiences. Another widely used term of ‘after-death communications’ did not seem appropriate because only some of the participants in this study felt that their experience was a specific communication with the deceased.

It is fair to disclose that the researcher that performed the analysis has not had similar experiences to those people studied, nor do they hold any strong prejudice about the phenomena that could have adversely influenced the process. Furthermore, the group of authors includes members with different prior knowledge of the phenomena, which has been taken to offer a diverse range of topic-related experience and new perspectives on the topic.

### Data Collection

The study focused on individuals who had experienced the death of a close one. The data consisted of 408 experience narratives offered by 181 participants and was collected during 2013–2020 through an electronic questionnaire. In addition to the participant’s background information, the questionnaire asked participants to describe the personal consequences of their unexplained experiences related to the death of a close one. The participants gave their informed consent for their narratives to be used for the purposes of this study. The participant data was handled confidentially and the study reported in a way that ensured the anonymity of the participants.

### Data Analysis

The study included narratives of unexplained experiences that had occurred either at the moment of a close one’s death, or at any time after it. The distribution of background variables was reviewed using SPSS version 27 software. The data was analysed through inductive content analysis, using Atlas-ti software. Relevant units of analysis were identified in the data. The original expressions were simplified, and simplifications with similar content were grouped into broad categories. The analysis formed categories and subcategories which were assigned names that comprehensively described their contents. The overall data became more structured during the analysis, but the original information was still retained. The original quotations were revisited at different stages of the analysis (Bengtsson, 2016), and the resulting article has been written using the Standards for Reporting Qualitative Research (SRQR) – checklist produced by the EQUATOR-network to support the quality of reporting (O'Brien et al., 2014).

## Results

### Research Participants

All of the participants in the study were female because no males responded to the study request. The average age of the participants was 44 (Table 1), the youngest being 16 years old and the oldest 69. Most participants described their health as good or very good (70%). The majority of participants were employed or studying (71%), had at least an upper secondary education (68%), were in a relationship (57%), and belonged to a religious denomination (81%). The majority of respondents (72%) reported a period of less than 5 years since the death of a close one. The majority (71%) of participants reported having more than one unexplained experience, but only 28% of them described more than two different types of experiences.

**Table 1.** Background Variables of the Participants.

Background variable	*N*	Valid %
Health		
Very good	21	11.6
Fairly good	106	58.6
Moderate	44	24.3
Fairly bad or very bad	10	5.6
Work participation		
Full-time employment	91	51.2
Part-time employment	13	7.3
Unemployment/furlough	9	5.1
Retired	14	7.9
Sick leave	10	5.6
Stay-at-home parent/caregiver	19	10.7
Student	22	12.4
Education level		
Basic education	9	5.0
Vocational training	18	9.9
Vocational diploma	42	23.2
Secondary level vocational degree	54	29.8
University of applied Sciences degree	28	15.5
University degree	30	16.6
Marital status		
Married	74	40.9
In a domestic partnership	29	16.0
Single	23	12.7
Divorced/separated	22	12.2
Widowed	33	18.2
Religious denomination		
Evangelical Lutheran	143	78.6
Other	5	2.7
None	33	18.1
Time passed since the death at the time of participation		
Less than a year	40	22.1
1–5 years	91	50.3
6–10 years	26	14.4
More than 10 years	24	13.3

Regarding the deceased, 64% were men and 36% women (Table 2), and 60% were over 18 years of age at the time of their death, with an average age of 32. As regards their relationship with the bereaved individual, the deceased were either children or grandchildren (56%), spouses or ex-spouses (24%), siblings or friends (12%) or parents or grandparents (8%). A majority of the deceased had passed away due to illness (44%), but other causes of death were suicide (21%), accident (19%), and stillbirth or cot death (13%). In the majority of cases (60%), the death had been sudden.

**Table 2.** Background Variables of the Deceased Person.

Background variables	*N*	Valid %
Gender of the deceased		
Male	116	64.1
Female	65	35.9
Age of death		
Foetal stage	25	13.1
Infancy (less than 1 year)	11	7.2
Childhood (1–12 years)	22	12.4
Adolescence (13–17 years)	12	6.8
Early adulthood (18–29 years)	40	22.6
Adulthood (30–49 years)	38	21.5
Late adulthood (50–69 years)	24	13.6
Old age (over 70 years)	5	2.8
The role of the deceased in the participant’s life		
Child or grandchild	102	56.4
Spouse or ex-spouse	44	24.3
Sibling or close friend	21	11.6
Parent or grandparent	14	7.7
Cause of death		
Illness	80	44.0
Accident	34	18.7
Stillbirth/cot death	24	13.2
Suicide	38	20.9
Homicide	4	2.2
Unknown at the time of participation	2	1.1
Sudden versus expected death		
Sudden	108	59.7
Expected	73	40.3

### The Consequences of Unexplained Experiences for Bereaved Individuals

As consequences of unexplained experiences, the bereaved reported experiencing after-effects, and consequences related to sharing and documenting the experience (Table 3).

**Table 3.** Consequences of Unexplained Experiences.

Aspect of consequence	Outcomes
After-effects	Alleviating consequences for wellbeing
	Aggravating consequences for wellbeing
	Life-affecting changes
Consequences related to sharing the experience	Recounting the experience
	Concealing the experience
	The reactions of loved ones upon recounting the experience
Documenting the experience	Keeping mementos
	Filming the phenomenon
	Photographing the phenomenon

## After-Effects

The after-effects of unexplained experiences included alleviating and aggravating consequences for wellbeing, as well as life-affecting changes. Alleviating consequences for wellbeing were described as emotions that supported coping, lifted the mood and lowered stress levels. They also involved gaining a confirmation of the deceased’s wellbeing, positive thoughts on the experience, and positive experiences of bonding.

Emotions that supported coping were a sense that everything will be okay, a sense of getting help, getting comfort, easing the work of grief and reducing guilt. They also included hopefulness, a stronger belief in the future and empowerment. Emotions that lifted the mood involved feeling good, joyful and happy. Emotions that lowered stress levels included having a warm and peaceful feeling, feeling lighter, feeling relaxed and having an increased feeling of safety.

‘The experience was lovely and comforting. It felt like solace was pouring down on me from heaven’.

‘I was left with a enormous feeling of peace of which I often recall’.

‘The experience created a feeling of safety, and that my spouse lets me know that he has been visiting our dogs and me’.

Gaining confirmation of the deceased’s wellbeing involved feeling that the deceased was well and free of suffering, feeling that the deceased was in a good place, and also having a feeling of reconciliation with the deceased. Positive thoughts on the experience included gratitude for the experience and a desire for new experiences. Positive experiences of bonding involved the experience of having a continuous bond with the deceased and with God.

‘He was free, painless and happy, and he expressed it to me through those events’.

‘I am grateful to him for sharing this experience with me and allowing me to experience it’.

‘I am grateful for all these experiences. It feels good when [the deceased] is in some way present here at home and during our trips together’.

Aggravating consequences for wellbeing included emotions that lowered the mood and raised stress levels. Emotions that lowered the mood involved an increased longing, a feeling of letting go, sadness and powerlessness. Emotions that raised stress levels were a fear of the experience recurring, and a general fear of the supernatural.

‘The dreams triggered a deep longing, but also a feeling of letting go’.

‘I’m now pregnant for the first time since the death of [the deceased], and I fear that the experience will happen again’.

Life-affecting changes involved changes to one’s outlook on life and one’s actions. Changes to one’s outlook on life involved an increased appreciation for life, an increased interest in unexplained phenomena, an easier acceptance of death, a reduced fear of death, increased lovingness in one’s worldview, and a stronger belief in reunion and an afterlife. Changes to one’s actions included avoiding certain places, and following the instructions of the deceased.

‘I lost my fear of death, my mind is full of images of this beautiful, happy place’.

‘But the experiences have helped me to understand that there is life after death’.

‘For some time, I was scared to go to the building site of our new home alone’.

### Consequences Related to Sharing the Experience

Consequences related to sharing an unexplained experience involved recounting the experience, concealing the experience, and the reactions of loved ones upon recounting the experience. Recounting the experience involved discussing it either openly or selectively. Concealing the experience involved choosing not to share it due to the personal nature of the experience, and also due to the fear of being stigmatised as having mental issues. The reactions of loved ones included the loved ones’ concern for the bereaved individual, and also their belief in the authenticity of the experience.

‘These are things I feel and know exist for certain, but I wouldn’t tell anyone about them. They would think I’m mental’.

‘I was left with a wonderful and peaceful feeling. And I found myself telling others about it with excitement, and didn’t think of it as a paranormal experience’.

‘When I told my mother about the experience she was terrified’.

‘Afterwards, I told my husband about what happened and he told me he had experienced the same thing’.

### Documenting the Experience

As a consequence of the experience, bereaved individuals kept mementos, and filmed and photographed the phenomenon.

‘We never found out where the feathers came from. My husband and I kept one of them’.

‘I once got the encounter on video’.

‘I ran out to get my camera and took a picture, and the clouds vanished’.

## Discussion

According to the findings of this study, unexplained experiences in the context of bereavement generate both alleviating and aggravating consequences, where some previous studies have described them as positive experiences (Beischel et al., 2014; Field et al., 2013; Foster et al., 2011; Jahn & Spencer-Thomas, 2014; Steffen & Coyle, 2011). The emotions associated with the letting go of a loved one and the emotions that raise stress levels can be linked to the higher levels of anxiety or more difficult grief reactions that have been reported in previous literature (Boulware & Bui, 2016; Currier et al., 2015; Field & Friedrichs, 2004; Sirrine et al., 2018). The results of this study support Foster et al.’s (2011) and Klass and Steffen’s (2018) observations that such experiences are not unambiguously either beneficial or harmful to the bereaved individual and that consequences vary across individuals. This study showed that unexplained experiences have significant consequences that promote the coping of the bereaved individual, such as an increased coping ability, and emotions that lift the mood and lower stress levels. The experience of having a continuing bond with the deceased and also gaining a confirmation of their wellbeing illustrates the significance of a post-death relationship to the bereaved individual. The positive thoughts evoked by the experience support the view that unexplained experiences in the context of bereavement are not necessarily to be seen as scary, and in fact, their recurrence may even be desired, which has not been reported in previous studies. Since many bereaved individuals reported experiencing both alleviating and aggravating consequences simultaneously, the consequences of the experience can be regarded as being more relevant to the provision of support than the internalised or externalised nature of the experience. Thus, due to the complex nature of the phenomenon, research that combines both qualitative and quantitative methods is required.

The results of the study indicated changes in one’s perspective on life, such as a more positive outlook on life, a reduced fear of death, and a stronger belief in an afterlife and reunion, which supports the results of previous studies (Keen et al., 2013). The bereaved individuals tended to regard these changes in perspective on life as positive and also felt that the changes contributed to coping with grief and daily life. For some bereaved individuals, these changes in perspective related to a strengthening of existing beliefs, while others experienced radical changes in their beliefs about an afterlife, which has also been stated by Keen et al. (2013). A radical change in life perspective may require a restructuring of one’s worldview, and it is therefore important that bereaved individuals receive the support they need during this process. The power of such experiences is illustrated by their effect on the actions of the people who have experienced them, such as avoiding certain places as a result of the experience, or even following the instructions provided by the deceased during the experience. Thus, this study suggests that such experiences may have a very concrete impact on the lives of bereaved individuals and may cause the behaviour of the bereaved individuals to appear illogical if they do not have a possibility to safely process their experiences.

The consequences of unexplained experiences for bereaved individuals are diverse, and based on the results of this study, having a possibility to process such experiences with professionals seems important. These experiences also have long-term effects on bereaved individuals, so gradually processing them after bereavement may promote coping. Healthcare professionals should offer information to bereaved individuals about the possibility of such experiences and also create an atmosphere that encourages them to discuss them (Steffen & Coyle, 2011). The normalisation of such experiences is important because according to Harper et al., (2011), socially accepted CB manifestations are more beneficial for the coping of the bereaved than those that may raise social stigma. Consequently, increasing people’s knowledge surrounding the normality of such experiences would also support the loved ones of bereaved individuals, as in the study findings, some loved ones were concerned for the wellbeing of the bereaved person after they had shared their experience.

What is essential regarding the possibility of sharing such experiences is receiving support in order to process their consequences. Processing them at an emotional level is also important, since according to Field et al. (2013), one’s experience of the nature of the situation will have an impact on its consequences. Furthermore, the need of the bereaved to document their experiences (which emerged in this study) may also indicate a need to validate the experience. Keeping objects that were found during the experience may also be seen as a symbolic representation of a post-death relationship, and according to Harper et al. (2011), accompanying symbolic representations are linked to a better coping with grief. Thus, their conscious use in support interventions for bereaved individuals should be investigated.

As a final point, it should be recognised that a limitation to this study is that all of the participants were female. This was not seen as a major issue because of the qualitative descriptive research approach taken. However, in future, it would be interesting to study whether these experiences are more common among females than males, or whether it could simply be that females are more willing to share their experiences in a study.

## Conclusion

Unexplained experiences affect bereaved individuals in various ways and can either promote or complicate coping. The empowering consequences of such experiences should be reinforced through support interventions aimed at bereaved individuals. The impact of the experiences can also lead to radical changes in the person’s worldview, and the bereaved individuals often need support in dealing with the changes that take place. Accordingly, the awareness of healthcare professionals regarding unexplained experiences and their consequences related to the death of a close one should be increased in order to avoid the unnecessary pathologising of the experiences and to enable their processing in a safe environment.

### Relevance for Clinical Practice

This study offers new information regarding the consequences of unexplained experiences relating to the death of a close one. Despite the fact that these experiences are fairly common among bereaved individuals, the phenomenon is not widely known among healthcare professionals. Consequently, healthcare professional’s better understanding of unexplained experiences and their consequences will encourage them to discuss these themes with those who are grieving, and offer them a broader level of social support. The increased knowledge of this phenomenon can further normalise these unexplained experiences related the death of a close one and so reduce the stigma that is related to them. Ultimately, this may reduce the fear and confusion of bereaved people who face unexplained experiences related to the death of a close one.
